# Agrobacteria deploy two classes of His-Me finger superfamily nuclease effectors exerting different antibacterial capacities against specific bacterial competitors

**DOI:** 10.3389/fmicb.2024.1351590

**Published:** 2024-02-14

**Authors:** Mary Nia M. Santos, Katherine L. Pintor, Pei-Yu Hsieh, Yee-Wai Cheung, Li-Kang Sung, Yu-Ling Shih, Erh-Min Lai

**Affiliations:** ^1^Institute of Plant and Microbial Biology, Academia Sinica, Taipei, Taiwan; ^2^Molecular and Biological Agricultural Sciences Program, Taiwan International Graduate Program, National Chung-Hsing University and Academia Sinica, Taipei, Taiwan; ^3^Graduate Institute of Biotechnology, National Chung-Hsing University, Taichung, Taiwan; ^4^Aquaculture Research and Development Division, Department of Agriculture-National Fisheries Research and Development Institute (DA-NFRDI), Manila, Philippines; ^5^Institute of Biological Chemistry, Academia Sinica, Taipei, Taiwan; ^6^Biotechnology Center, National Chung-Hsing University, Taichung, Taiwan

**Keywords:** type VI secretion system (T6SS), interbacterial antagonism, bacterial toxin, nuclease, *Agrobacterium*

## Abstract

The type VI secretion system (T6SS) assembles into a contractile nanomachine to inject effectors across bacterial membranes for secretion. The *Agrobacterium tumefaciens* species complex is a group of soil inhabitants and phytopathogens that deploys T6SS as an antibacterial weapon against bacterial competitors at both inter-species and intra-species levels. The *A. tumefaciens* strain 1D1609 genome encodes one main T6SS gene cluster and four *vrgG* genes (i.e., *vgrGa-d*), each encoding a spike protein as an effector carrier. A previous study reported that *vgrGa-*associated gene 2, named *v2a,* encodes a His-Me finger nuclease toxin (also named HNH/ENDO VII nuclease), contributing to DNase-mediated antibacterial activity. However, the functions and roles of other putative effectors remain unknown. In this study, we identified *vgrGc-*associated gene 2 (*v2c*) that encodes another His-Me finger nuclease but with a distinct Serine Histidine Histidine (SHH) motif that differs from the AHH motif of V2a. We demonstrated that the ectopic expression of V2c caused growth inhibition, plasmid DNA degradation, and cell elongation in *Escherichia coli* using DNAse activity assay and fluorescence microscopy. The cognate immunity protein, V3c, neutralizes the DNase activity and rescues the phenotypes of growth inhibition and cell elongation. Ectopic expression of V2c DNase-inactive variants retains the cell elongation phenotype, while V2a induces cell elongation in a DNase-mediated manner. We also showed that the amino acids of conserved SHH and HNH motifs are responsible for the V2c DNase activity *in vivo* and *in vitro*. Notably, V2c also mediated the DNA degradation and cell elongation of the target cell in the context of interbacterial competition. Importantly, V2a and V2c exhibit different capacities against different bacterial species and function synergistically to exert stronger antibacterial activity against the soft rot phytopathogen, *Dickeya dadantii*.

## Introduction

The type VI secretion system (T6SS) is a nanomachine used by many Gram-negative bacteria for antagonism or pathogenesis by injecting toxins into target bacterial or eukaryotic cells (Hachani et al., 2016; Hood et al., 2017; Coulthurst, 2019). T6SS is composed of a membrane complex in connection to a baseplate, which is the docking site for the polymerization of a tube surrounded by a contractile sheath (Wang et al., 2019). The tube is a puncturing device stacked of a Hcp protein hexamer tipped with the trimeric valine–glycine repeat protein G (VgrG) spike and a PAAR repeat-containing protein, which sharpens the tip (Shneider et al., 2013). When the sheath contracts, the puncturing device carrying effectors is propelled toward the target cell to deliver effectors. After firing, the sheath disassembles, and the subunits of the sheath are recycled to build another machine (Basler et al., 2012).

In terms of delivery, the T6SS effectors can be classified as cargo or specialized effectors. The cargo effectors interact non-covalently with Hcp, VgrG, or PAAR, while in specialized effectors, the effector domain is covalently fused to Hcp, VgrG, or PAAR (Cianfanelli et al., 2016). Functionally, most of the identified effectors mediate antibacterial activities (e.g., nuclease, peptidoglycan hydrolase, lipase, phospholipase, NAD (P) + −glycohydrolase, and ADP-ribosyltransferase) by targeting conserved cellular structures such as nucleic acids, peptidoglycan, and the inner membrane, as well as specific metabolites or proteins (Lien and Lai, 2017; Cherrak et al., 2019).

To date, the characterized T6SS nuclease effectors include members of the HNH, N-Tox, Tox-Rease, and PoNe superfamilies and were shown to target DNA substrates without specificity (Ma et al., 2014, 2017; Fitzsimons et al., 2018; Pissaridou et al., 2018; Fridman et al., 2020; Pei et al., 2020; Santos et al., 2020). Thus, the expression of these T6SS nuclease effectors causes DNA degradation *in vitro* and/or *in vivo*. Interestingly, the majority of T6SS DNase effectors belong to the HNH/ENDO VII nuclease superfamily, also known as the His-Me finger endonuclease superfamily, consisting of 38 distinct families (Jablonska et al., 2017; Wu et al., 2020). These nuclease superfamily proteins harbor three conserved His-Asp-His within an approximately 30-amino acid domains formed by a β1 hairpin followed by an α-helix to form a ββα-metal topology for DNA binding and hydrolysis (Jablonska et al., 2017; Wu et al., 2020). Several T6SS effectors belonging to this HNH/His-Me superfamily, including Tse7 of *Pseudomonas aeruginosa* as Tox-GHH2 (Pissaridou et al., 2018) and Tse1 of *Aeromonas dhakensis* (Pei et al., 2020), V2a of *Agrobacterium tumefaciens* as Tox-AHH (Santos et al., 2020), and two Tox-SHH family effectors, Tke4 of *Pseudomonas putida* (Bernal et al., 2017) and Txe4 of the fish pathogen *Pseudomonas plecoglossicida* (Li et al., 2022). Amino acid substitution experiments on Tse7, V2a, and Tse1 have indicated that this conserved catalytic site [A/G] HH is required for nuclease activity and toxicity (Pissaridou et al., 2018; Pei et al., 2020; Santos et al., 2020). However, the impacts of SHH and HNH motifs of the Tox-SHH family effectors on nuclease activity and T6SS toxicity have not been demonstrated.

*Agrobacterium tumefaciens* is a Gram-negative Alphaproteobacteria belonging to the *Rhizobiaceae* family. It is an economically important pathogen that causes crown gall disease and a gene delivery tool with the capability of transforming plant cells and fungi (Hwang et al., 2017). The T6SS main gene cluster is highly conserved in the genome of the *A. tumefaciens* genomospecies complex (Wu et al., 2019, 2021; Chou et al., 2022). Besides the main cluster consisting of two divergent operons, *imp* (impaired in nitrogen fixation) encode core structural and regulatory components and *hcp* operon for effectors and effector delivery components (e.g., Hcp, VgrG, and PAAR). Most *A. tumefaciens* strains also encode additional *vgrG* operons encoding effector-immunity (EI) gene pairs. The *A. tumefaciens* strain 1D1609 encodes four *vgrG* genes (*vgrGa, vgrGb, vgrGc,* and *vgrGd*) with each *vgrG* module associated with three to four associated genes, named as *vgrG-associated* genes 1–4 in cluster *a/b/c/d* (Santos et al., 2020). Each *vgrG* gene is genetically linked to a conserved chaperone/adaptor gene and different known or putative EI pairs, including three effectors with a conserved N-terminal PAAR-like DUF4150 domain but distinct C-terminal effector domains (V2a, V2c, and V2d) (Santos et al., 2020; Supplementary Figure S1). For the *vgrGb* module, V2b does not encode an effector domain but downstream two genes encode a putative ADP-ribosylating enzyme (V3b) and a Rhs-linked effector domain (V4b), followed by putative immunity. A previous study by deleting single or multiple EI pairs suggested that *vgrGa-associated* effector V2a, a DNase effector harboring the C-terminal Tox-AHH domain, appears to be the major antibacterial toxin, but the *vgrGd-associated* effector V2d also contributes to full antibacterial activity for 1D1609 against *E. coli* prey (Santos et al., 2020). The biochemical and biological functions of other putative effectors encoded in the other three orphan *vgrG* modules remain uncharacterized.

In this study, we discovered that the *vgrGc-*associated effector V2c is a Tox-SHH DNase toxin and explored the rationale for having two T6SS DNase toxins in *A. tumefaciens* strain 1D1609. We show that the expression of V2c causes growth inhibition, plasmid DNA degradation, and cell elongation, and that these phenotypes are neutralized by the co-expression of the cognate immunity protein, V3c. We also show that the SHH motif is responsible for the nuclease activity of V2c. Importantly, V2a AHH nuclease and V2c SHH nuclease exert different antibacterial capacities against specific bacterial competitors and function synergistically to exert stronger antibacterial activity against the soft rot phytopathogen, *Dickeya dadantii*. Harboring two classes of His-Me finger nuclease effectors with potential target-specific toxicity may grant 1D1609 versatile antibacterial weapons in facing different bacterial competitors in the microbial community.

## Materials and methods

### Bacterial strains and growth conditions

Bacterial strains and plasmids are listed in Supplementary Table S1. *E. coli* strains were grown in Luria-Bertani (LB) medium at 37°C, *A. tumefaciens* strains in 523 medium at 25°C, and *D. dadantii* 3937 in LB medium at 30°C. Antibiotics were added when necessary: for *E. coli*, 30 μg/mL of gentamicin and 250 μg/mL of streptomycin and for *D. dadantii* and *A. tumefaciens*, 50 μg/mL of gentamicin.

### Molecular cloning

Plasmid pJN105 was used for the expression of toxin genes, and the pTrc200 plasmid was used for the expression of immunity genes. PCR was performed with KAPA HiFi Hot Start DNA Polymerase (Roche, Switzerland) using the genomic DNA of *A. tumefaciens* 1D1609. The primers used in the study are listed in Supplementary Table S2. Site-directed mutagenesis was performed using either DpnI or Gibson Assembly (New England BioLabs, USA), ligated using T4 ligase (Takara), and transformed into chemically competent *E. coli* DH10B. Plasmid DNA was isolated using the QIAprep Miniprep Kit (Qiagen, Germany). All constructs were confirmed by sequencing. The in-frame deletion mutants in *A. tumefaciens* were generated using a suicide plasmid via double crossing over by electroporation or by conjugation, as described previously (Santos et al., 2020).

### Bioinformatic analysis

The orthologs of V2c were analyzed using amino acid sequences after the DUF4150 domain via BLASTP search. Multiple sequence alignment was performed using MUltiple Sequence Comparison by Log-Expectation (MUSCLE) (Edgar, 2004). Full-length sequences of the V2c orthologs were analyzed to obtain domain architectures from the Pfam database (Mistry et al., 2021). The *E*-value threshold was set at 10^−5^. HHpred (Soding et al., 2005) and Swiss Model (Waterhouse et al., 2018) were used for homology-based protein structure prediction. Protein structure was predicted by AlphaFold (Jumper et al., 2021; Varadi et al., 2022) from the UniProt database. Secondary structure prediction was performed using default settings in PSIPRED 4.0 Protein Structure Prediction Server/DMPfold 1.0 Fast Mode (Buchan and Jones, 2019; Greener et al., 2019).

### Growth inhibition assay

The growth inhibition assay was performed as described previously (Ma et al., 2014). In brief, overnight cultures of the *E. coli* DH10B strain with empty vectors or the derivatives were adjusted to an OD_600_ of 0.1 in LB medium. A measure of 1 mM isopropyl β-D-1-thiogalactopyranoside (IPTG, Amresco) was used to induce the expression of the putative immunity protein. After 1 h of IPTG induction, L-arabinose (Ara, Sigma-Aldrich) was added to the final concentration of 0.2% to induce the expression of the toxin. Cell growth was recorded every hour at OD_600_. Empty vectors were used as controls. After 8 h, cells were harvested to quantify the number of colony-forming units (log_10_ CFU/mL) by automatic diluter and plater, easySpiral Dilute (Interscience, France).

### Plasmid DNA degradation analysis in *Escherichia coli* cells

*In vivo* plasmid DNA degradation analysis was performed as described previously (Ma et al., 2014). In brief, overnight cultures of the *E. coli* DH10B strain with empty vectors or the derivatives grown in LB broth were harvested and adjusted to OD_600_ of approximately 0.3 in LB medium. *E. coli* cultures were induced with 1 mM IPTG at 0 h for *v3c* expressed from the pTrc200 plasmid, followed by L-arabinose (0.2%, final concentration) induction at 1 h to induce *v2c* from the pJN105 plasmid, and cultured for 2 h. Equal amounts of cells were used for plasmid DNA extraction, and an equal volume of extracted DNA was resolved in agarose gel, followed by ethidium bromide staining, and visualized using Gel Doc XR+ UV Gel Documentation Molecular Imager Universal Hood II (Bio-Rad, USA).

### Cloning, expression, and purification of V2c and mutants

For the *in vitro DNase* activity assay, the effector gene *v2c* was cloned in the pET28a plasmid to generate N-terminal His-tagged V2c, and the immunity gene *v3c* was constructed in the pTrc200 plasmid. Site-directed mutagenesis was performed by using overlap extension PCR. The plasmid for expressing effector protein, *v2c* or its variants, and the plasmid for expressing immunity protein, *v3c*, were transformed into *E. coli* BL21 (DE3) for IPTG-induced co-expression. The bacterial culture was incubated at 37°C until OD_600_ reached 0.5 and then 0.1 mM IPTG was added, followed by 5-h expression at 37°C. Pellets were collected, sonicated, and expressed proteins were purified using Ni Sepharose 6 Fast Flow histidine-tagged protein purification resin (Cytiva, Germany). Briefly, cells were lysed in extraction buffer (50 mM Tris–Cl, pH7.5, 0.1 M NaCl, 20 mM imidazole), and proteins were eluted by using an elution buffer with up to 250 mM imidazole in 50 mM Tris–Cl, pH7.5, 0.1 M NaCl.

### *In vitro* DNase activity assay

A 10-μL reaction mixture containing 200 ng of pTrc200HA plasmid DNA and 1× CutSmart Buffer (New England BioLabs, USA) with partially purified V2c or its variants normalized by the major V2c protein band was incubated at 37°C for 1 h. The digestion of DNA was determined by agarose gel electrophoresis and visualized by SYBR Safe DNA gel stain (Invitrogen, USA). The reaction samples of V2c or its variants were scaled up for Western blotting analysis. An anti-V2c antibody was generated in rabbits against two V2c peptides, PFYWDFPNSQVGRD and KRIAMLRGDPTRYD (indicated in Supplementary Figure S2), by Yao-Hong Biotechnology Inc. (Taiwan).

### Western blotting analysis

A Western blotting analysis was performed as described previously (Lai and Kado, 1998). In general, proteins were resolved by SDS-PAGE and transferred onto a PVDF membrane by using a transfer apparatus (Bio-Rad, USA). The membrane was probed with primary antibodies against V2c (1:4000), Hcp (1:2500), TssB (1:4000), and GFP (1:1000), followed by incubation with a horseradish peroxidase-conjugated anti-rabbit secondary antibody (ChemiChem) (1:25000) and visualized with the ECL system (Perkin-Elmer Life Science, Boston, USA).

### Fluorescence microscopy and image analysis

*Escherichia coli* DH10B carrying the empty vectors or the derivatives were grown overnight in LB broth at 37°C. For the expression of the effector gene alone, overnight culture was diluted to OD_600_ reached 0.2 and sub-cultured for 1.5 h prior to induction by 0.2% L-arabinose for 1 h. For the co-expression of effector and putative immunity genes, overnight culture was diluted to OD_600_ reached 0.1 and sub-cultured for 1 h prior to induction by 0.2% L-arabinose and 1 mM IPTG for 3–4 h. Cultures were harvested and resuspended with 30 μL of PBS with 0.5% Tween 20 (PBST). Three μL of cells were placed on a 2% agarose pad and imaged. To check the DNA degradation, the same protocol was followed, and live cells were stained with 2 μg/mL of Hoechst (Life Technologies, USA) and 1 μg/mL of FM4-64FX, a fixable analog of FM 4–64 (Invitrogen, USA) for 5 min and washed with PBS to remove the excess dye.

For the co-culture of *D. dadantii* ∆*imp*-GFP (S65T) with various 1D1609 EI pair mutants, strains were mixed at 1:10 ratio on Agrobacterium kill-triggering (AK) agar and the AK medium (3 g K_2_HPO_4_, 1 g NaH_2_PO_4_, 1 g NH_4_Cl, 0.15 g KCl, 9.76 g MES in 900 mL of ddH_2_0, pH 5.5) (Yu et al., 2020) was solidified by 2% (w/v) agar. Cells from 16-h co-culture at 25°C were concentrated to an OD_600_ of 10 and spotted on a 2% agarose pad for imaging.

Imaging was performed using an inverted fluorescent microscope (IX81, Olympus Japan) with the objective lens UPLSAPO 100XO (Olympus, Tokyo, Japan), DAPI (49,000; Chroma), GFP (49,002; Chroma), and Cy3 (49,005; Chroma) filter sets and a CCD camera (ORCA-R2; Hamamatsu, Japan). The images were acquired using Olympus cellSens Dimension software.

All the image analysis was performed using the Fiji analyze particles tool (Schindelin et al., 2012). The micrograph of FM 4–64 fluorescence was used to segment surfaces (i.e., individual bacterial cells) with background subtraction and thresholding. Feret diameter was used. The cell length (μm) was determined from the cumulative counts of three random frames per sample. For the quantification of cell degradation, the Hoechst intensity was normalized using the formula intensity/unit area of cells, where intensity is major × minor × 3.14 (π).

### Interbacterial competition assay

Bacterial competitions were carried out as described previously (Wu et al., 2019) with modifications. Briefly, an overnight culture of *A. tumefaciens* and *D. dadantii* 3937 ∆*imp* was mixed at a 10:1 attacker-to-prey ratio and spotted on AK agar plates (Yu et al., 2020). The prey, *A. tumefaciens* C58 or *D. dadantii* 3937 ∆*imp*, was transformed with pRL662-GFP (S65T) for gentamicin selection or microscopy imaging. After 18 h of incubation at 25°C, the co-cultured cells were collected, serially diluted, and plated on LB agar containing gentamicin to quantify surviving prey cells by counting CFU.

### Statistical analysis

The statistical analyses were performed using GraphPad Prism version 6.01 for Windows, GraphPad Software, La Jolla, California, USA.^1^ The number of technical replicates and independent biological replicates, *p*-values, and statistical tests performed are indicated in the figure legends. The mean of independent replicates was compared using the one-way ANOVA followed by Tukey’s multiple comparisons tests^2^ for statistical analysis. The error bars indicate the standard error of the mean (SEM).

## Results

### V2c is a DNase belonging to the Tox-SHH clade of His-Me finger superfamily

**Figure 1:**
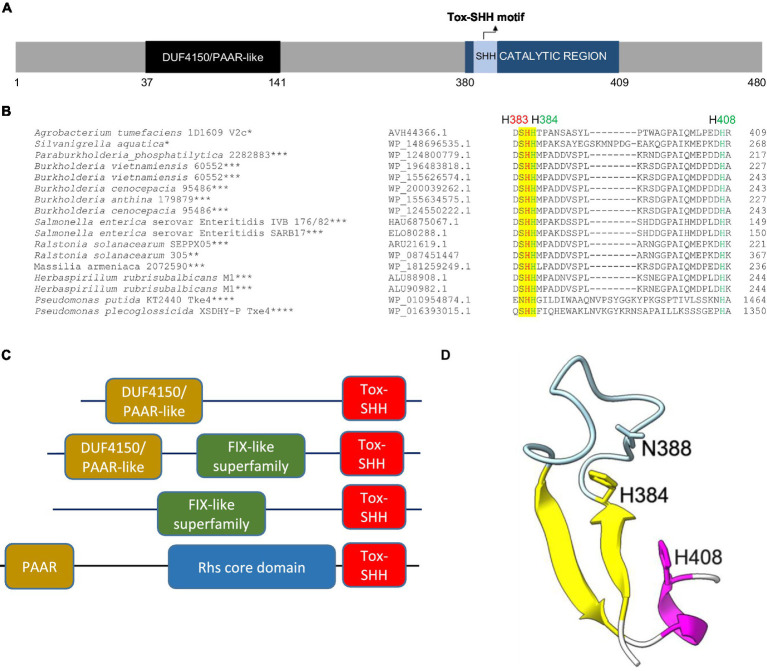
V2c is a PAAR-linked specialized effector containing a C-terminal Tox-SHH motif. **(A)** A schematic diagram of V2c protein showing the N-terminal DUF4150 PAAR region and the catalytic region (amino acids 380–409), which covers the Tox-SHH motif. **(B)** Partial sequence alignment of orthologs of V2c from BLASTP. The strain name and accession number are indicated on the left. SHH motif is highlighted in yellow, with H383 (indicated in red) as the metal ion coordinating residue, and H384 and H408 (indicated in green) of the HNH motif for catalysis and metal ion binding, respectively, are targeted for mutagenesis. The star corresponds to the specific domains shown in panel **(C)**: *DUF4150/PAAR-like, **DUF4150/PAAR-like + FIX-like, ***FIX-like superfamily, ****PAAR + Rhs core domain. **(C)** Domain architectures of V2c orthologs and Tox-SHH-containing proteins found in this study. Full-length sequences were analyzed, and the domains were from the Pfam database with *E* value threshold set at 10^−5^. **(D)** A cartoon model of V2c (residues 380–409). The structure of V2c predicted by AlphaFold indicates V2c that consists of a His-Me finger domain with two antiparallel β-strands (yellow) connected with an Ω loop (light blue), a histidine (H384) at the C-terminus of the first β-strand and is followed by an α-helix (magenta). The confidence score of this region is higher than 90, indicating high structure accuracy.

In 1D1609, *vgrGa*-associated effector V2a appears to be the major antibacterial toxin against *Escherichia coli* prey (Santos et al., 2020). V2c is a putative PAAR (DUF4150)-linked specialized effector (Figure 1A), but the presence or absence of *v2c* did not affect the antibacterial activity of 1D1609 against *E. coli* prey (Santos et al., 2020). Aside from the N-terminal PAAR domain, V2c does not show any sequence similarity to V2a (Supplementary Figure S2). BLAST and CDD searches did not identify a known or conserved domain at its C-terminal region. However, multiple sequence alignments of V2c and V2c orthologs revealed conserved SHH and HNH motifs at the C-terminal region (Figure 1B). These V2c orthologs all harbor the C-terminal Tox-SHH domain, but their N-terminal region can be fused to other known T6SS domains, such as Found in the type sIX effector (FIX), the DUF4150/PAAR-like domain, and the Rhs core domain (Jana et al., 2019; Figure 1C). Using the protein homology detection program HHpred (Soding et al., 2005), we found that the C-terminal region of V2c encodes two parallel β-strands connected by an α-helix, which is a signature structure in His-Me finger nuclease (Wu et al., 2020). Consistent with the result of secondary structure prediction, 3D structure prediction using AlphaFold (Jumper et al., 2021; Varadi et al., 2022; Figure 1D) shows that this domain consists of two antiparallel β-strands connected with an Ω loop with a His (H384) at the C-terminus of the first β-strand and followed by an α-helix, featuring a HNH motif of His-Me finger endonuclease (Jablonska et al., 2017; Wu et al., 2020). These suggest that V2c belongs to the Tox-SHH (Pfam PH15652) clade of the His-Me finger superfamily.

**Figure 2:**
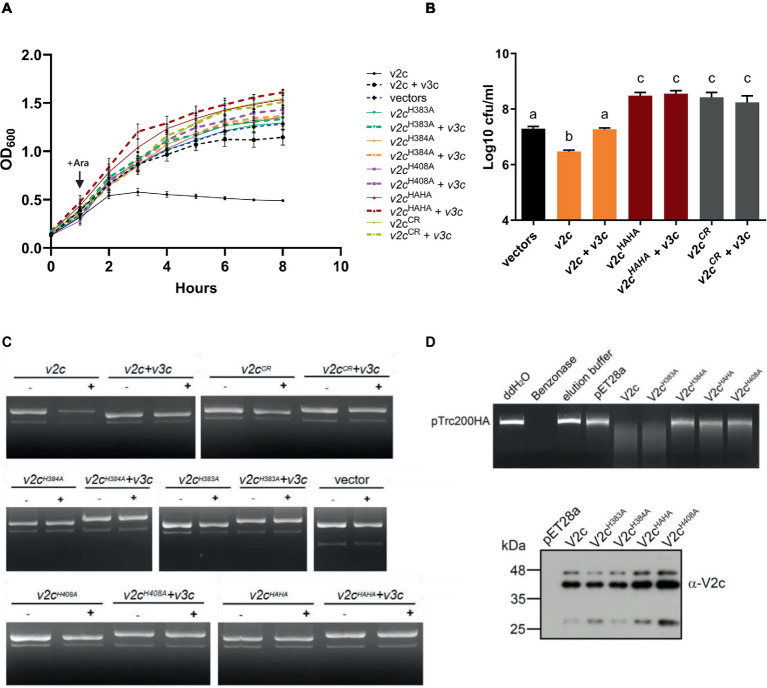
V2c exhibits growth inhibition and DNase activity *in vivo* and *in vitro.* **(A)** Growth inhibition analysis of expression of *v2c* and its variants with or without *v3c* in *E. coli* DH10B. *E. coli* cultures were induced with 1 mM IPTG at 0 h for *v3c* expressed from the pTrc200 plasmid, followed by L-arabinose (Ara) induction at 1 h to induce *v2c* from the pJN105 plasmid. Cell growth was recorded every hour at OD_600_. Strain expressing empty vectors (vectors) were used as a control. **(B)** Number of colony-forming units (log_10_ CFU/mL) quantified after growth inhibition assay at 8 h. Data in panel **(A,B)** represent mean ± SEM of four independent experiments (*n* = 4), each averaged with three technical repeats. Different letters above the bar indicate statistically different groups of strains (*p* < 0.05) determined by Tukey’s HSD test. **(C)** Plasmid degradation assay performed by *E. coli* DH10B cells harboring pTrc200 and pJN105 plasmids (vector) or the derivatives induced with (+) or without (−) Ara for 2 h. Plasmid DNA was extracted, and the degradation pattern was observed in agarose gel. **(D)** *In vitro* DNase activity assay performed by using pTrc200HA plasmid DNA as DNA substrate and incubated with purified V2c or its variants. Nuclease-free water (ddH_2_O), protein elution buffer (elution buffer), and eluate from empty vector expression (pET28a) served as negative controls, while Benzonase nuclease served as a positive control. The digestion of the DNA substrate was determined by agarose gel electrophoresis. The reaction samples of V2c or its variants were detected by Western blotting using an anti-V2c antibody (α-V2c). The results of panel **(C,D)** are representative of three independent experiments.

To determine the antibacterial and DNase activities of V2c, we expressed it using an arabinose-inducible promoter to determine if V2c is a bacterial toxin functioning as a nuclease. Growth inhibition measured by optical density was observed when *v2c* is expressed by arabinose induction in *E. coli*, and this can be neutralized by the co-expression of its putative cognate immunity, *v3c*, as compared to the vector control (Figure 2A). Furthermore, the colony-forming unit (CFU) count shows the reduced CFU of *v2c-*expressed cells, and higher CFU could be recovered with the co-expression of *v3c* (Figure 2B). In addition, the plasmid degradation assay showed that the expression of *v2c* upon arabinose induction resulted in the degradation of plasmid DNA in *E. coli*, and this is abolished when *v3c* is co-expressed (Figure 2C). The data indicated that V2c exhibits DNase activity *in vivo*.

We further investigated whether the SHH and HNH motifs are involved in DNase activity. The His-Me finger superfamily has a strictly conserved His residue and catalytic metal ion, which are both essential for nucleic acid hydrolysis. Based on this premise, we define a region spanning amino acid residues 380–409 as a His-Me finger domain core region (CR). Based on the alignment, H384 is the catalytic His and H408 is for metal ion binding, while H383 may function as a second residue for metal ion coordination. To verify their roles in antibacterial and DNase activity, a series of deletions as well as single and double amino acid substitution variants were generated. Strikingly, no growth inhibition or plasmid degradation were observed in any of the mutants expressing the CR deletion (V2c^CR^), single (V2c^H383A^, V2c^H384A^, and V2c^H408A^), or double (V2c^HAHA^ substitution in both H383 and H384) variants (Figures 2A,C,D). Higher CFU was also recovered from *E. coli* culture expressing these mutants alone or with *v3c* (Figure 2B). To further confirm the DNase activity of V2c, each V2c and its variants were fused with 6x-His and purified for *in vitro* DNase activity assay. The results showed that the purified V2c fraction is able to degrade DNA but not the eluate from *E. coli* expressing the vector (pET28a) (Figure 2D). Consistent with the *in vivo* plasmid degradation assay, no activity could be detected from V2c^H384A^, V2c^H408A^, or V2c^HAHA^ variants. However, the purified V2c^H383A^ fraction exhibited DNase activity by degrading the plasmid DNA substrate. The Western blotting analysis of the reaction samples shows whether V2c and its variants used for the assay were at similar protein levels. Taken together, the results of *in vivo* and *in vitro* DNA degradation assays show that the conserved H384 and H408 in the HNH motif are crucial for V2c DNase activity, whereas H383 in the SHH motif appears to be less critical for *in vitro* but may be required for *in vivo* nuclease activity.

### Ectopic expression of V2c caused DNA degradation and cell elongation

**Figure 3:**
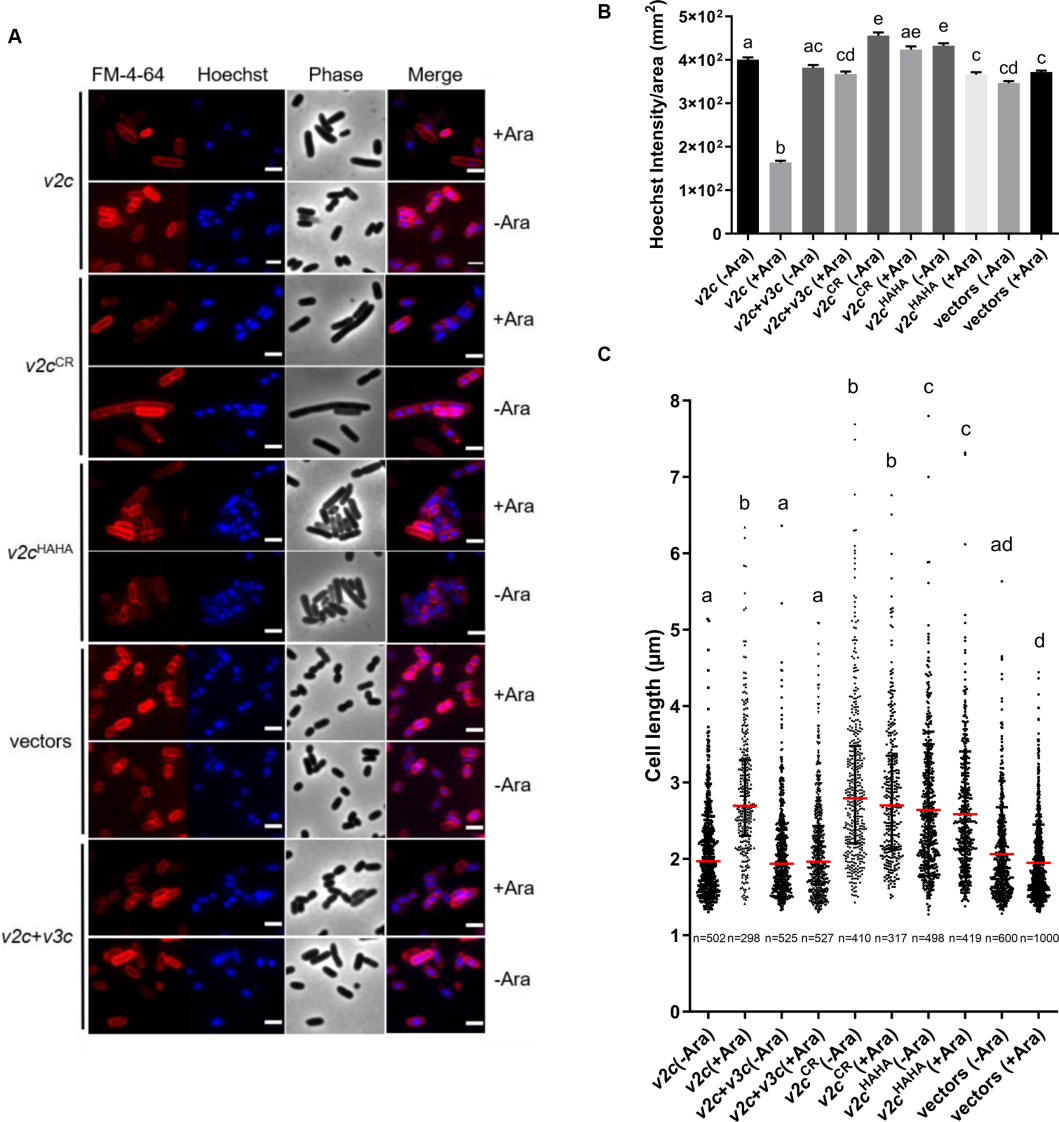
V2c Tox-SHH DNase exhibits the cell elongation phenotype. **(A)** Images of *E. coli* DH10B cells harboring vector(s) and the derivatives expressing *v2c* or its variants in the presence or absence of its cognate immunity gene *v3c* with (+Ara) and without (-Ara) Arabinose induction stained with FM 4–64 (red) and Hoechst stain (blue). The micrographs from left to right are FM 4–64, Hoechst, phase contrast, and a merged image of FM 4–64 and Hoechst stains. Scale bar, 2 μm. Representative images of two independent experiments are shown. **(B)** Normalized Hoechst intensity (intensity/unit area of cells) and **(C)** cell length (μm) as measured from the indicated number of cells (n) per treatment. The graph shows a combined count from three frames of a representative result of two independent experiments. The red line in panel **(C)** shows the median with an interquartile range. Statistics were performed with the mean ± SEM of three frames. Different letters above the bar indicate statistically different groups of strains (*p* < 0.05) determined by Tukey’s HSD test.

We next examined the cell morphology and DNA integrity of *E. coli* cells expressing *v2c* and the two variants (*v2c^CR^* and *v2c^HAHA^*) that are defective in DNase activity and toxicity. Cell morphology was observed by phase contrast, while the bacterial cell membrane and DNA were stained by FM 4–64 and Hoechst, respectively, under the fluorescence microscope. Hoechst signals are significantly reduced when *v2c* is expressed by arabinose induction in *E. coli*, and this can be rescued by the co-expression of its cognate immunity *v3c* shown in the images and quantification (Figures 3A,B). Hoechst signals are diminished only in *E. coli* cells producing *v2c* but not *v2c*^HAHA^, *v2c*^CR^, and vector control, suggesting that cellular DNA degradation is caused by V2c DNase activity, which can be neutralized by its immunity protein V3c. In addition, we also observed cell elongation in *E. coli* expressing *v2c*, and co-expression of *v3c* rescues the cell elongation phenotype (Figures 3A,C). However, *E. coli* cells expressing *v2c*^CR^ and *v2c*^HAHA^, as well as other single amino acid substitution variants (*v2c^H383A^*, *v2c^H384A^*, and *v2c^H408A^*), do not abolish the elongated cell phenotype (Figures 3A,C; Supplementary Figures S3A,B).

### Ectopic expression of V2a also caused cell elongation phenotype

**Figure 4:**
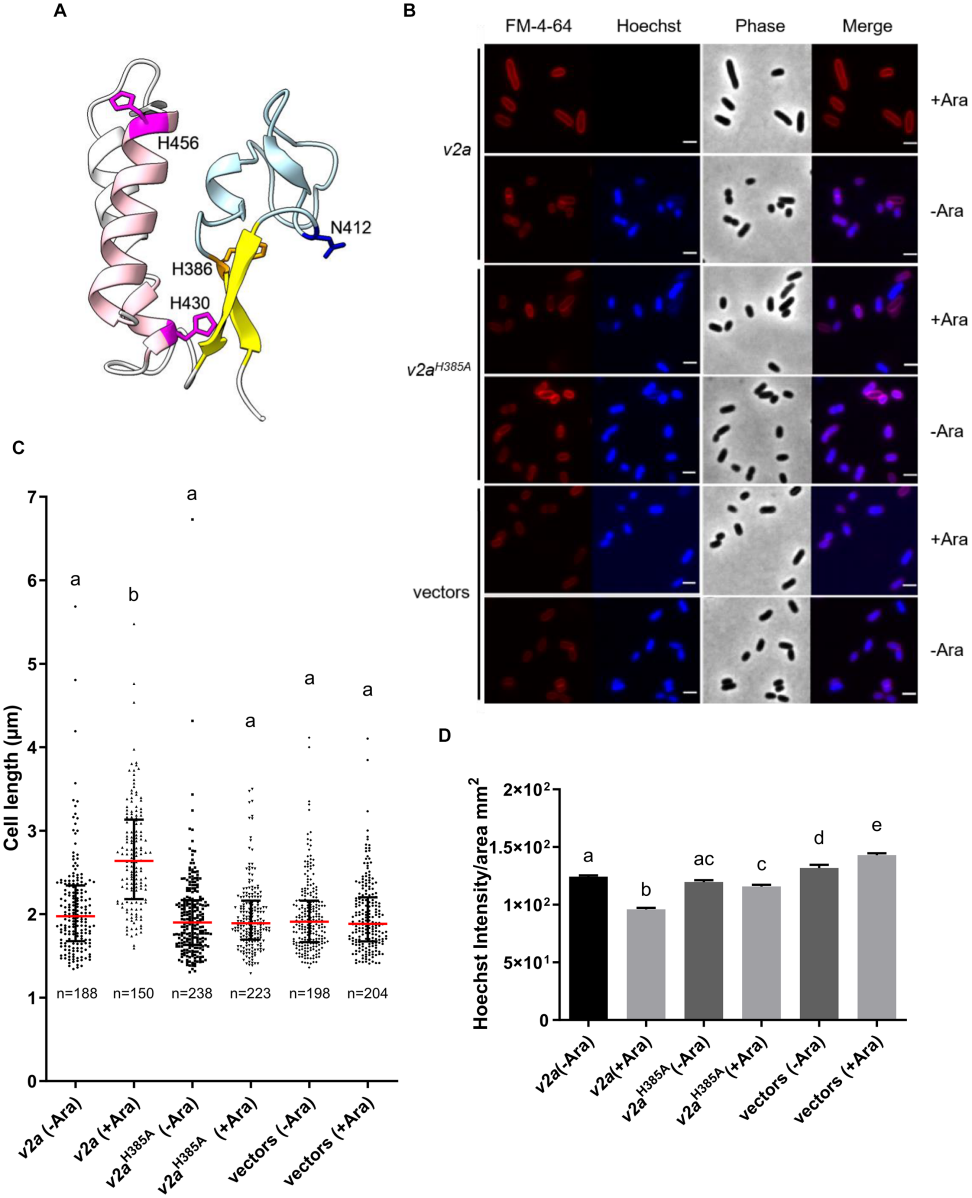
V2a Tox-AHH DNase exhibits the cell elongation phenotype. **(A)** A cartoon model of V2a (residues 380–470). The structure of V2a predicted by AlphaFold indicates V2a consisting of a His-Me finger domain with its signature antiparallel β-strands (yellow) connected with an Ω loop (light blue) with a histidine (H386, orange) at the C-terminus of the first β-strand. Two α-helices (pink) with histidine residues (H430, H456 magenta) followed the β-strands. N412 is found in the loop between the β-strands, and H430 is in the first α-helix (E429–G430). The predicted local-distance difference tests (pLDDTs) of the His-Me domains of V2a range from confident to very high; most of the pLDDTs of the residues are >90. **(B)** Morphological analysis of *E. coli* DH10B cells harboring vector(s) or its derivatives expressing *v2a*, catalytic site (H385A) mutant without (-Ara) or with (+Ara) arabinose induction. Cells were stained with FM 4–64 (red) and Hoechst stain (blue). The micrographs from left to right are FM 4–64, Hoechst, phase contrast, and a merged image of the two fluorescent images. Scale bar, 2 μm. **(C)** Cell length (μm) and **(D)** Hoechst intensity in different treatments as determined from a combined count of three random frames of a representative result; the number of cells (n) per sample is indicated. The graph shows a combined count from three frames of a representative result of two independent experiments. The red line shows the median with an interquartile range. Statistics were performed with the mean ± SEM of three frames. Different letters above the bar indicate statistically different groups of strains (*p* < 0.05) determined by Tukey’s HSD test.

A previous study showed that V2a is a Tox-AHH nuclease that serves as the major antibacterial weapon in 1D1609 (Santos et al., 2020). H385 and H386 are two of the predicted His residues in the AHH motif of V2a, corresponding to H383 and H384 in the SHH motif of V2c. The structure predicted by AlphaFold indicates that V2a also features a His-Me finger, having two α-helices with histidine residues (H430 and H456) followed by the β-strands (Figure 4A). Previous findings demonstrated that the expression of *v2a* but not *v2a^H385A^* caused growth inhibition and plasmid degradation in *E. coli* (Santos et al., 2020). To gather further insight into the V2a AHH motif in the His-Me finger domain, we evaluated the growth and morphology of arabinose-induced *v2a* mutants. The results showed that the expression of *v2a* caused a reduction in Hoechst-stained DNA signals and resulted in cell elongation (Figures 4B–D). The expression of *v2a^H385A^* has similar levels of Hoechst-stained DNA signals and cell length as compared to the vector control. These results suggest that the cell elongation phenotype observed was caused by V2a DNase activity, whereas V2c shows DNase activity-independent cell elongation. We also determined the toxicity of another putative effector V4b of 1D1609, a PAAR (DUF4150)-linked specialized effector but harboring an Rhs domain with structural similarity to the insecticidal toxin (Santos et al., 2020; Supplementary Figure S4A). Expression of *v4b* caused growth inhibition in *E. coli,* in which the growth inhibition can be rescued by the co-expression of its putative cognate immunity *v5b* to a level similar to vector control (Supplementary Figure S4B). However, no DNA degradation or cell elongation could be observed (Supplementary Figures S4C–F). These results show that V4b is a bacterial toxin and V5b is the cognate immunity protein, suggesting that the cell elongation phenotype may be specific to the toxicity of nuclease effectors.

### V2c nuclease exhibits antibacterial activity at both intra-species and inter-species competition and functions synergistically with V2a against the soft rot phytopathogen, *Dickeya dadantii*

**Figure 5:**
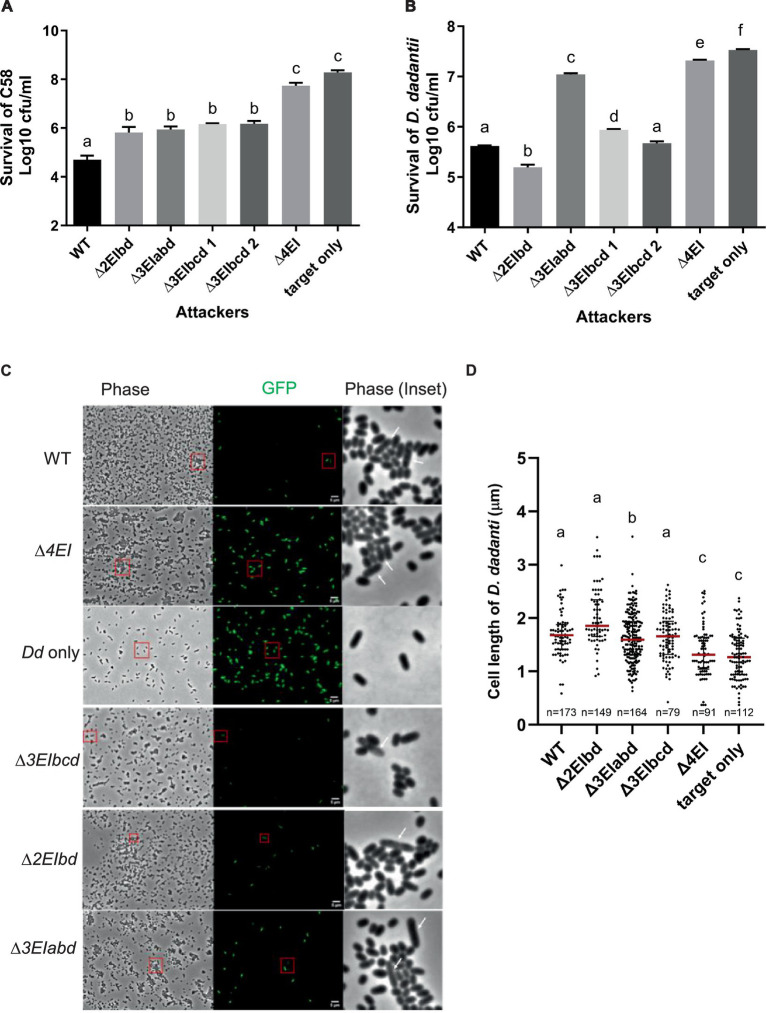
V2a and V2c exhibit different toxicity against *A. tumefaciens* and *D. dadantii*. **(A)** Intra-species competition between *A. tumefaciens* 1D1609 attacker and prey C58. **(B–D)** Inter-species competition between *A. tumefaciens* 1D1609 attacker and prey *D. dadantii* 3937 ∆*imp.* The plasmid pRL-GFP(S65T) plasmid (Gm^R^) was transformed into prey cells, which were mixed at a 1:10 ratio with each of the attacker strains, 1D1609 WT, or various EI mutant strains as indicated. After co-culture on AK agar plates, cells were collected, serially diluted, and plated on the LB agar plate containing Gm for CFU counting of surviving prey cells **(B)**. Data are mean ± SEM (*n* = 6 biological repeats from three independent experiments). For imaging, co-cultured *D. dadantii* Δ*imp*-GFP(S65T) and 1D1609 EI pair mutants were concentrated and spotted on a 2% agarose pad for fluorescence micrographs **(C)**. *D. dadantii* Δ*imp*-GFP(S65T) indicated as Dd only is included as a control. The inset in phase contrast shows a magnified image of cells in the red box. White arrows point to *D. dadantii* cells. Scale bar, 2 μm. **(D)** Cell length after competition between *A. tumefaciens* 1D1609 EI mutants and *D. dadantii* 3937 ∆*imp*-GFP(S65T). Cell length of GFP fluorescent *D. dadantii* cells was measured using the Fiji analyze particles tool. Cell length (μm) in different treatments as determined from a combined count of at least 10 random frames of two independent experiments. The red line shows the median with an interquartile range. Statistics were performed with the mean ± SEM of total counts. Different letters above the bar indicate statistically different groups of strains (*p* < 0.05) determined by Tukey’s HSD test.

To correlate the DNA degradation and cell elongation phenotypes observed by the ectopic expression of *v2c* in *E. coli* in a more biologically relevant context, we further performed the interbacterial competition assay. We first selected *A. tumefaciens* strain C58, which possesses incompatible EI pairs with 1D1609 and has previously been demonstrated to be susceptible to 1D1609 T6SS killing (Wu et al., 2019), for intra-species competition. Each of the 1D1609 mutants lacking multiple EI pairs was co-cultured with a C58 strain harboring pRL-GFP (S65T) prey *in vitro* on AK agar (Yu et al., 2020) and the number of viable C58 prey cells (log_10_ CFU/mL) was quantified. Similar to the previous killing assay using *E. coli* prey (Santos et al., 2020), we detected approximately 2 log of reduced prey cell survival by the 1D1609 WT attacker as compared to the *∆4EI* mutant with the deletion of all four EI pairs (Figure 5A). Other double (*∆EIbd*) or triple deletion mutants (*∆EIabd, ∆EIbcd*) exhibited compromised but detectable antibacterial activity against C58, indicating that *v2a* or *v2c* alone is sufficient to exhibit antibacterial activity against C58.

Intrigued by the observation that *v2c* could exhibit antibacterial activity to the *A. tumefaciens* C58 prey but no significant effect against *E. coli* prey (Santos et al., 2020), we reasoned that *E. coli* may not be a physiologically relevant prey for *A. tumefaciens* although *v2c* is able to cause toxicity when directly expressed in *E. coli* (Figures 2A,B). We then selected *D. dadantii* as the prey cells for inter-species competition because it is a phytopathogen but also a close relative of *E. coli*, both belonging to *Enterobacteriaceae*. To avoid potential counterattack by *D. dadantii* T6SS (Koskiniemi et al., 2013), a T6SS-deficient mutant, ∆*imp* with deletion of *imp* operon, harboring pRL-GFP (S65T) (*D. dadantii ∆imp*-GFP (S65T)) was used as a prey. CFU counting of *D. dadantii ∆imp*-GFP (S65T) prey cells after co-culture show approximately 1.5 log of reduced prey cell survival by 1D1609 WT attacker, as compared to the *∆4EI* mutant (Figure 5B). The presence of *v2a* only (*∆3EIbcd*) exhibited comparable antibacterial activity as the WT, while *v2c* only (*∆3EIabd*) showed weak but detectable antibacterial activity as compared to ∆*4EI,* indicating that *v2c* itself is able to kill *D. dadantii* but with much weaker activity than *v2a*. Strikingly, 1D1609 expressing both *v2a* and *v2c* (*∆2EIbd*) could result in stronger killing effects than WT. The use of pRL-GFP (S65T) with constitutive expression of GFP also allowed us to observe the *D. dadantii ∆imp*-GFP (S65T) prey cells under the fluorescence microscope after co-culture. We observed very few cells expressing GFP when competing with WT or *∆3EIbcd* expressing *v2a* only. The lysed and elongated *D. dadantii* cells were also detected when co-cultured with 1D1609 with either strains expressing *v2a* only (*∆3EIbcd*), *v2c* only (*∆3EIabd*), or both *∆3EIbd* (Figure 5C). We further quantified the cell length of GFP-expressing *D. dadantii* cells. The results show that the majority of cells were elongated after 18 h of co-culture with 1D1609 expressing either *v2a* (*∆3EIbcd*) or *v2c* (*∆3EIabd*), or a combination of both (*∆3EIbd*), as compared to those *D. dadantii* cells alone or co-incubated with *∆4EI* (Figure 5D).

Taken together, these results suggest that V2c nuclease exhibits antibacterial activity at both intra-species and inter-species competitions but at different capacities. While V2a and V2c exert similar antibacterial activity against *A. tumefaciens* C58, V2c only exhibits weak antibacterial activity but functions synergistically with V2a when competing with *D. dadantii* prey, resulting in elongated and lysed cells.

## Discussion

His-Me finger nucleases are a large and diverse superfamily of nucleases; however, only a limited number of T6SS effectors have been identified as His-Me finger nucleases. In the present study, we reported that *A. tumefaciens* strain 1D1609 encodes two His-Me finger superfamily DNases, V2a and V2c, belonging to the Tox-AHH and Tox-SHH families, respectively, exhibiting different capacities for specific bacterial competitors. It is well established that the effector encoded in the *vgrG* module is delivered by its cognate VgrG carrier via T6SS (Hachani et al., 2014; Whitney et al., 2014; Bondage et al., 2016; Flaugnatti et al., 2016). Together with our previous studies that 1D1609 with deletion of four EI pairs or four *vgrG* genes completely loses the T6SS-mediated antibacterial activity (Santos et al., 2020), we concluded that the V2a and V2c are T6SS effectors, although direct demonstration is yet to be determined.

His-Me finger nucleases are one-metal-ion-dependent nucleases, primarily dependent on Mg^2+^ or, in some cases, on Zn^2+^. The only invariant residue in His-Me endonuclease is the catalytic His located at the end of the β1 strand (first H in the HNH motif, H384 for V2c, and H386 for V2a, Figures 1D, 4A; Supplementary Figure S2), which functions as the general base for the hydrolysis of phosphodiester bonds. The other conserved His (last H in the HNH motif, H408 for V2c and H430 for V2a) and Asn/Asp/Glu/His located before the invariant His (H383 for V2c and H384 for V2a) are involved in coordinating the single metal ion. Indeed, our data show that H383, H384, and H408 of V2c and H385 and H386 of V2a are required for exerting DNase activity *in vivo* (Figure 2C; Santos et al., 2020). However, different from the loss of DNase activity *in vivo* and *in vitro* for both V2c^H384A^ and V2c^H3408A^, the V2c^H383A^ variant retains the DNase activity by an *in vitro* nuclease activity assay (Figure 2D), which indicates the requirement of H383 in coordinating metal ion binding for DNase activity may be subject to environmental factors.

His-Me finger encodes a small structural motif, so it is usually associated with other domain architectures that could play additional functions. In addition, its three-element structural motif is unable to provide enough of a hydrophobic core for stability (Jablonska et al., 2017). Thus, many proteins containing the His-Me finger employ a variety of additional structural elements or domains for stabilization and/or specificity. V2c and its orthologs commonly found in *Rhizobiaceae* are not included in the reported 77 sequences with conserved core regions of the His-Me finger superfamily (Jablonska et al., 2017). Our evidence further suggests that V2c may represent a new branch of the Tox-SHH toxin family (Pfam PF15652), which includes proteins with additional domain architectures (Figure 1C). These additional domain(s) are conserved regions found in many bacterial toxin proteins and may facilitate the secretion of nuclease and affect its toxicity to the competing cell. While both V2c and V2a harbor the conserved N-terminal DUF4150 PAAR-like domain, they share low sequence similarity in the C-terminal His-Me finger domain (Supplementary Figure S2), in which the protein structures predicted by AlphaFold reveal the structural difference between V2c and V2a (Figures 1C, 4A). This discrepancy may contribute to the difference on toxicity strength and the role of DNase on cell elongation. Alternatively, V2a and V2c may have the same mechanism to bind and hydrolyze DNA for toxicity, and the difference in the phenotype is caused by another domain outside the His-Me finger fold.

Cell elongation could still be observed in the *E. coli* strain carrying different DNase-defective *v2c* variants, even in the absence of arabinose (Figures 3A,C; Supplementary Figure S3A). Several studies reported that the leaky expression of T6SS toxin driven by the arabinose-inducible P_BAD_ promoter resulted in toxicity in the absence of arabinose (Jana et al., 2019; Fridman et al., 2020). Thus, we speculated that the leaky expression of these *v2c* variants is sufficient to induce the cell elongation phenotype. The data suggest that another domain outside the SHH catalytic region in V2c is responsible for the cell elongation phenotype, which is different from the nuclease activity-dependent filamentation observed previously (Yu and Lai, 2017; Pissaridou et al., 2018) and V2a (Figure 4), suggesting that V2c-induced cell elongation is independent of nuclease activity. The proposed biological role of bacterial filamentation is mainly related to the stress response to thrive for survival (Justice et al., 2008). One of the conditions that lead to filamentation is DNA damage. When the DNA is damaged, the SOS response is induced, and cells may delay the cell cycle and inhibit cell division until DNA repair and replication are complete. Although V2c catalytic site variants lose activity for DNA cleavage, they may still bind to DNA and induce cell elongation. Furthermore, cell division arrest could also be caused by non-nuclease effectors. For example, a T6SS ADP-ribosyltransferase effector Tre1 in *Serratia proteamaculans* was reported to inactivate bacterial cell division by modifying arginine on FtsZ to block polymerization (Ting et al., 2018). A future study by domain dissection of V2c is needed to identify the domain causing cell elongation.

While HNH/His-Me T6SS nucleases are the most prevalent nuclease toxins identified in T6SS, most bacterial genomes only encode one type of HNH/His-Me nuclease effector, including Tse7 of *P. aeruginosa* (Pissaridou et al., 2018), Tse1 of *A. dhakensis* (Pei et al., 2020), and Tke4 of *P. putida* (Bernal et al., 2017). In addition to *A. tumefaciens* 1D1609 encoding Tox-AHH (V2a) and Tox-SHH (V2c) nucleases, the use of multiple HNH/His-Me nucleases as T6SS antibacterial weapons has been identified in the fish pathogen *P. plecoglossicida* (Li et al., 2022). *P. plecoglossicida* T6SS-2 mediates interbacterial competition and encodes four putative effectors, all of which contain C-terminal toxin domains belonging to the HNH/His-Me superfamily but with distinct classes (Txe1 with a dipeptide HH motif, Txe2 as Tox-AHH, Txe3 with HNHc, and Txe4 as Tox-SHH) (Li et al., 2022). Among them, Txe1, Txe2, and Txe4 exhibit *in vitro* nuclease activity and toxicity when expressed in *E. coli*. However, only Txe1 and Txe4 contribute to interbacterial activity against *E. coli,* with Txe1 as the predominant toxin, in which its C-terminal conserved dipeptide HH motif is required for nuclease activity and toxicity to *E. coli.* Txe4 is also required for full interbacterial competition, but the role of the SHH motif in DNase and toxicity has not been determined. Together with our findings, it is possible that having multiple distinct or the same classes of nuclease effectors may provide versatile toxicity when competing with different preys, in which some toxins may only exhibit activity against specific preys.

The strain 1D1609 is unique in the *A. tumefaciens* species complex with multiple VgrG spikes carrying different antibacterial effectors (Santos et al., 2020). Our findings that *v2a* and *v2c* exhibit different capacities against different preys support previous studies that some T6SS toxins can only exert toxicity in specific bacterial species (LaCourse et al., 2018; Wu et al., 2021). From the observation of the synergism of V2a and V2c against *D. dadantii*, harboring multiple T6SS effectors in 1D1609 may provide an advantage for the strain to maintain growth competitiveness in different niches and environments.

## Data availability statement

The original contributions presented in the study are included in the article/Supplementary material, further inquiries can be directed to the corresponding author.

## Author contributions

MS: Conceptualization, Investigation, Writing – original draft, Writing – review & editing, Methodology. KP: Investigation, Writing – review & editing, Methodology. P-YH: Investigation, Methodology, Writing – review & editing. Y-WC: Investigation, Methodology, Writing – review & editing. L-KS: Investigation, Methodology, Writing – review & editing. Y-LS: Funding acquisition, Investigation, Methodology, Resources, Supervision, Writing – review & editing. E-ML: Conceptualization, Funding acquisition, Investigation, Project administration, Resources, Supervision, Writing – original draft, Writing – review & editing.

## Footnotes

^1^ www.graphpad.com

^2^ http://astatsa.com/OneWay_Anova_with_TukeyHSD/

## References

[ref1] Basler M. Pilhofer M. Henderson G. P. Jensen G. J. Mekalanos J. J. (2012). Type VI secretion requires a dynamic contractile phage tail-like structure. Nature 483, 182–186. doi: 10.1038/nature10846, PMID: 22367545 PMC3527127

[ref2] Bernal P. Allsopp L. P. Filloux A. Llamas M. A. (2017). The *Pseudomonas putida* T6SS is a plant warden against phytopathogens. ISME J. 11, 972–987. doi: 10.1038/ismej.2016.169, PMID: 28045455 PMC5363822

[ref3] Bondage D. D. Lin J. S. Ma L. S. Kuo C. H. Lai E. M. (2016). VgrG C terminus confers the type VI effector transport specificity and is required for binding with PAAR and adaptor-effector complex. Proc. Natl. Acad. Sci. U. S. A. 113, E3931–E3940. doi: 10.1073/pnas.1600428113, PMID: 27313214 PMC4941472

[ref4] Buchan D. W. A. Jones D. T. (2019). The PSIPRED protein analysis workbench: 20 years on. Nucleic Acids Res. 47, W402–W407. doi: 10.1093/nar/gkz297, PMID: 31251384 PMC6602445

[ref5] Cherrak Y. Flaugnatti N. Durand E. Journet L. Cascales E. (2019). Structure and activity of the type VI secretion system. Microbiol. Spectr. 7. doi: 10.1128/microbiolspec.PSIB-0031-2019 PMC10957189 31298206

[ref6] Chou L. Lin Y. C. Haryono M. Santos M. N. M. Cho S. T. Weisberg A. J.. (2022). Modular evolution of secretion systems and virulence plasmids in a bacterial species complex. BMC Biol. 20:16. doi: 10.1186/s12915-021-01221-y, PMID: 35022048 PMC8756689

[ref7] Cianfanelli F. R. Monlezun L. Coulthurst S. J. (2016). Aim, load, fire: the type VI secretion system, a bacterial Nanoweapon. Trends Microbiol. 24, 51–62. doi: 10.1016/j.tim.2015.10.005, PMID: 26549582

[ref8] Coulthurst S. (2019). The type VI secretion system: a versatile bacterial weapon. Microbiology 165, 503–515. doi: 10.1099/mic.0.000789, PMID: 30893029

[ref9] Edgar R. C. (2004). MUSCLE: multiple sequence alignment with high accuracy and high throughput. Nucleic Acids Res. 32, 1792–1797. doi: 10.1093/nar/gkh340, PMID: 15034147 PMC390337

[ref10] Fitzsimons T. C. Lewis J. M. Wright A. Kleifeld O. Schittenhelm R. B. Powell D.. (2018). Identification of novel *Acinetobacter baumannii* type VI secretion system antibacterial effector and immunity pairs. Infect. Immun. 86:e00297-18. doi: 10.1128/IAI.00297-18, PMID: 29735524 PMC6056853

[ref11] Flaugnatti N. Le T. T. Canaan S. Aschtgen M. S. Nguyen V. S. Blangy S.. (2016). A phospholipase A1 antibacterial type VI secretion effector interacts directly with the C-terminal domain of the VgrG spike protein for delivery. Mol. Microbiol. 99, 1099–1118. doi: 10.1111/mmi.13292, PMID: 26714038

[ref12] Fridman C. M. Keppel K. Gerlic M. Bosis E. Salomon D. (2020). A comparative genomics methodology reveals a widespread family of membrane-disrupting T6SS effectors. Nat. Commun. 11:1085. doi: 10.1038/s41467-020-14951-4, PMID: 32109231 PMC7046647

[ref13] Greener J. G. Kandathil S. M. Jones D. T. (2019). Deep learning extends de novo protein modelling coverage of genomes using iteratively predicted structural constraints. Nat. Commun. 10:3977. doi: 10.1038/s41467-019-11994-0, PMID: 31484923 PMC6726615

[ref14] Hachani A. Allsopp L. P. Oduko Y. Filloux A. (2014). The VgrG proteins are "a la carte" delivery systems for bacterial type VI effectors. J. Biol. Chem. 289, 17872–17884. doi: 10.1074/jbc.M114.563429, PMID: 24794869 PMC4067218

[ref15] Hachani A. Wood T. E. Filloux A. (2016). Type VI secretion and anti-host effectors. Curr. Opin. Microbiol. 29, 81–93. doi: 10.1016/j.mib.2015.11.006, PMID: 26722980

[ref16] Hood R. D. Peterson S. B. Mougous J. D. (2017). From striking out to striking gold: discovering that type VI secretion targets bacteria. Cell Host Microbe 21, 286–289. doi: 10.1016/j.chom.2017.02.001, PMID: 28279332 PMC6404758

[ref17] Hwang H. H. Yu M. Lai E. M. (2017). Agrobacterium-mediated plant transformation: biology and applications. Am. Soc. Plant Biol. 15:e0186. doi: 10.1199/tab.0186 PMC6501860 31068763

[ref18] Jablonska J. Matelska D. Steczkiewicz K. Ginalski K. (2017). Systematic classification of the his-me finger superfamily. Nucleic Acids Res. 45, 11479–11494. doi: 10.1093/nar/gkx924, PMID: 29040665 PMC5714182

[ref19] Jana B. Fridman C. M. Bosis E. Salomon D. (2019). A modular effector with a DNase domain and a marker for T6SS substrates. Nat. Commun. 10:3595. doi: 10.1038/s41467-019-11546-6, PMID: 31399579 PMC6688995

[ref20] Jumper J. Evans R. Pritzel A. Green T. Figurnov M. Ronneberger O.. (2021). Highly accurate protein structure prediction with AlphaFold. Nature 596, 583–589. doi: 10.1038/s41586-021-03819-2, PMID: 34265844 PMC8371605

[ref21] Justice S. S. Hunstad D. A. Cegelski L. Hultgren S. J. (2008). Morphological plasticity as a bacterial survival strategy. Nat. Rev. Microbiol. 6, 162–168. doi: 10.1038/nrmicro1820, PMID: 18157153

[ref22] Koskiniemi S. Lamoureux J. G. Nikolakakis K. C. T'Kint de Roodenbeke C. Kaplan M. D. Low D. A.. (2013). Rhs proteins from diverse bacteria mediate intercellular competition. Proc. Natl. Acad. Sci. USA 110, 7032–7037. doi: 10.1073/pnas.1300627110, PMID: 23572593 PMC3637788

[ref23] LaCourse K. D. Peterson S. B. Kulasekara H. D. Radey M. C. Kim J. Mougous J. D. (2018). Conditional toxicity and synergy drive diversity among antibacterial effectors. Nat. Microbiol. 3, 440–446. doi: 10.1038/s41564-018-0113-y, PMID: 29459733 PMC5876133

[ref24] Lai E. M. Kado C. I. (1998). Processed VirB2 is the major subunit of the promiscuous pilus of *Agrobacterium tumefaciens*. J. Bacteriol. 180, 2711–2717. doi: 10.1128/JB.180.10.2711-2717.1998, PMID: 9573157 PMC107224

[ref25] Li Y. Yan X. Tao Z. (2022). Two type VI secretion DNase effectors are utilized for interbacterial competition in the fish pathogen *Pseudomonas plecoglossicida*. Front. Microbiol. 13:869278. doi: 10.3389/fmicb.2022.869278, PMID: 35464968 PMC9020831

[ref26] Lien Y. W. Lai E. M. (2017). Type VI secretion effectors: methodologies and biology. Front. Cell. Infect. Microbiol. 7:254. doi: 10.3389/fcimb.2017.00254, PMID: 28664151 PMC5471719

[ref27] Ma L. S. Hachani A. Lin J. S. Filloux A. Lai E. M. (2014). *Agrobacterium tumefaciens* deploys a superfamily of type VI secretion DNase effectors as weapons for interbacterial competition in planta. Cell Host Microbe 16, 94–104. doi: 10.1016/j.chom.2014.06.002, PMID: 24981331 PMC4096383

[ref28] Ma J. Pan Z. Huang J. Sun M. Lu C. Yao H. (2017). The Hcp proteins fused with diverse extended-toxin domains represent a novel pattern of antibacterial effectors in type VI secretion systems. Virulence 8, 1189–1202. doi: 10.1080/21505594.2017.1279374, PMID: 28060574 PMC5711352

[ref29] Mistry J. Chuguransky S. Williams L. Qureshi M. Salazar G. A. Sonnhammer E. L. L.. (2021). Pfam: the protein families database in 2021. Nucleic Acids Res. 49, D412–D419. doi: 10.1093/nar/gkaa913, PMID: 33125078 PMC7779014

[ref30] Pei T. T. Li H. Liang X. Wang Z. H. Liu G. Wu L. L.. (2020). Intramolecular chaperone-mediated secretion of an Rhs effector toxin by a type VI secretion system. Nat. Commun. 11:1865. doi: 10.1038/s41467-020-15774-z, PMID: 32313027 PMC7170923

[ref31] Pissaridou P. Allsopp L. P. Wettstadt S. Howard S. A. Mavridou D. A. I. Filloux A. (2018). The *Pseudomonas aeruginosa* T6SS-VgrG1b spike is topped by a PAAR protein eliciting DNA damage to bacterial competitors. Proc. Natl. Acad. Sci. U. S. A. 115, 12519–12524. doi: 10.1073/pnas.1814181115, PMID: 30455305 PMC6298103

[ref32] Santos M. N. M. Cho S. T. Wu C. F. Chang C. J. Kuo C. H. Lai E. M. (2020). Redundancy and specificity of type VI secretion *vgrG* loci in antibacterial activity of *Agrobacterium tumefaciens* 1D1609 strain. Front. Microbiol. 10:3004. doi: 10.3389/fmicb.2019.03004, PMID: 31993035 PMC6971182

[ref33] Schindelin J. Arganda-Carreras I. Frise E. Kaynig V. Longair M. Pietzsch T.. (2012). Fiji: an open-source platform for biological-image analysis. Nat. Methods 9, 676–682. doi: 10.1038/nmeth.2019, PMID: 22743772 PMC3855844

[ref34] Shneider M. M. Buth S. A. Ho B. T. Basler M. Mekalanos J. J. Leiman P. G. (2013). PAAR-repeat proteins sharpen and diversify the type VI secretion system spike. Nature 500, 350–353. doi: 10.1038/nature12453, PMID: 23925114 PMC3792578

[ref35] Soding J. Biegert A. Lupas A. N. (2005). The HHpred interactive server for protein homology detection and structure prediction. Nucleic Acids Res. 33, W244–W248. doi: 10.1093/nar/gki408 15980461 PMC1160169

[ref36] Ting S. Y. Bosch D. E. Mangiameli S. M. Radey M. C. Huang S. Park Y. J.. (2018). Bifunctional immunity proteins protect bacteria against FtsZ-targeting ADP-Ribosylating toxins. Cell 175, 1380–1392.e14. doi: 10.1016/j.cell.2018.09.037, PMID: 30343895 PMC6239978

[ref37] Varadi M. Anyango S. Deshpande M. Nair S. Natassia C. Yordanova G.. (2022). AlphaFold protein structure database: massively expanding the structural coverage of protein-sequence space with high-accuracy models. Nucleic Acids Res. 50, D439–D444. doi: 10.1093/nar/gkab1061, PMID: 34791371 PMC8728224

[ref38] Wang J. Brodmann M. Basler M. (2019). Assembly and subcellular localization of bacterial type VI secretion systems. Ann. Rev. Microbiol. 73, 621–638. doi: 10.1146/annurev-micro-020518-115420, PMID: 31226022

[ref39] Waterhouse A. Bertoni M. Bienert S. Studer G. Tauriello G. Gumienny R.. (2018). SWISS-MODEL: homology modelling of protein structures and complexes. Nucleic Acids Res. 46, W296–W303. doi: 10.1093/nar/gky427, PMID: 29788355 PMC6030848

[ref40] Whitney J. C. Beck C. M. Goo Y. A. Russell A. B. Harding B. De Leon J. A.. (2014). Genetically distinct pathways guide effector export through the type VI secretion system. Mol. Microbiol. 92, 529–542. doi: 10.1111/mmi.12571, PMID: 24589350 PMC4049467

[ref41] Wu C. C. Lin J. L. J. Yuan H. S. (2020). Structures, mechanisms, and functions of His-Me finger nucleases. Trends Biochem. Sci. 45, 935–946. doi: 10.1016/j.tibs.2020.07.002, PMID: 32807610

[ref42] Wu C. F. Santos M. N. M. Cho S. T. Chang H. H. Tsai Y. M. Smith D. A.. (2019). Plant-pathogenic *Agrobacterium tumefaciens* strains have diverse type VI effector-immunity pairs and vary in in-planta competitiveness. Mol Plant Microbe Int. 32, 961–971. doi: 10.1094/MPMI-01-19-0021-R, PMID: 30830835

[ref43] Wu C. F. Weisberg A. J. Davis E. W. 2nd Chou L. Khan S. Lai E. M.. (2021). Diversification of the type VI secretion system in Agrobacteria. MBio 12:e0192721. doi: 10.1128/mBio.01927-21, PMID: 34517758 PMC8546570

[ref44] Yu M. Lai E. M. (2017). Warfare between host immunity and bacterial weapons. Cell Host Microbe 21, 3–4. doi: 10.1016/j.chom.2016.12.012, PMID: 28081441

[ref45] Yu M. Wang Y. C. Huang C. J. Ma L. S. Lai E. M. (2020). *Agrobacterium tumefaciens* deploys a versatile antibacterial strategy to increase its competitiveness. J. Bacteriol. 203:e00490-20. doi: 10.1128/JB.00490-20 PMC7811202 33168638

