# Supplementary material for: Agrobacteria deploy two classes of His-Me finger superfamily nuclease effectors exerting different antibacterial capacities against specific bacterial competitors
Source: Front Microbiol. 2024 Feb 14;15:1351590. doi: 10.3389/fmicb.2024.1351590 (PMC10902643; doi:10.3389/fmicb.2024.1351590)
Supplement: Supplementary file 1 [file Data_Sheet_1.pdf]

Supplementary Material

Agrobacteria deploy two classes of His-Me finger superfamily nuclease effectors exerting different antibacterial capacities against specific bacterial competitors

Mary Nia M. Santos, Katherine L. Pintor, Pei-Yu Hsieh, Yee-Wai Cheung, Li-Kang Sung, Yu-Ling Shih and Erh-Min Lai*

*** Correspondence:** emlai@gate.sinica.edu.tw

Supplementary Figures

Supplementary Figure 1. Genetic loci of *A. tumefaciens* 1D1609 T6SS gene cluster and *vgrG* genetic modules. The four *vgrG* genes (*vgrGa*, *vgrGb*, *vgrGc*, *vgrGd*) and their associated effector-immunity pairs are indicated on linear chromosome. The number of *vgrG*-associated genes (v) represents the position to vgrG as defined previously [11].

Supplementary Figure 2. Sequence alignment of V2a and V2c. The conserved amino acid residues are indicated as '∗' (identical), ':' (similar) and '.' (slightly similar). The DUF4150, PAAR-like domain is in green. The histidine residues in AHH and SHH motifs in His-Me finger domain of V2a and V2c respectively, V2aH385/V2cH383 and V2aH386/V2cH384, are in red. The histidine residues that predicted to responsible for metal binding in His-Me finger domain, V2aH430/V2cH408, are in a bold blue font. Two peptides used for generating antibodies were underlined.

Supplementary Figure 3. Cell length analysis of *E. coli* DH10B cells expressing *v2c* catalytic site variants. (A) Expression of *v2c* single catalytic site variants. (B) Expression of catalytic site variants co-expressed with *v3c*. Cell length (μm) as measured from the indicated number of cells (n) per sample. The graph shows a combined count from six random frames of a representative result, the number of cells (n) per sample is indicated. The graph shows a combined count from 6 random frames, 3 from each of two independent experiments. The red line shows the median with interquartile range. Statistics were performed with data mean ± SEM of 6 frames. Different letters above the bar indicate statistically different group of strains ($P < 0.05$) determined by Tukey's HSD test. The red line shows the median with interquartile range.

Supplementary Figure 4. V4b exhibits growth inhibition, which could be neutralized by V5b. (A) Schematic diagram of V4b. The Rhs domain is bordered by conserved motifs. The CT is the C-terminal domain encoding a putative muramidase toxin. (B) *E. coli* growth inhibition analysis. *E. coli*

DH10B cultures were induced with 1 mM IPTG at 0 hour for the expression of putative immunity gene *v5b* from pTrc200 plasmid followed by L-arabinose (Ara) induction at 1 hour to induce putative *v4b* toxin gene from pJN105 plasmid. Cell growth was recorded every hour at OD600. DH10B expressing empty vectors were used as a control. Data are mean ± SD of three biological replicates. Similar results were observed in three independent experiments. (C) Plasmid degradation assay performed by using full length V4b (*v4b*FL) and C-terminal domain only (*v4b*CT). *E. coli* cells with pTrc200 and pJN105 plasmid (vectors) or expressing *v4b* and *v4b*+*v5b* were induced with (+) or without (-) arabinose (Ara) for 2 hour. Plasmid DNA was extracted and the degradation pattern was observed in agarose gel. Result shown is a representative data from two independent experiments. (D) Images of *E. coli* DH10B cells expressing *v4b* and *v4b* co-expressed with its cognate immunity gene *v5b* (*v4b*+*v5b*) under condition with (+Ara) or without (-Ara) arabinose induction. *E. coli* DH10B cells expressing empty vectors (vectors) under condition with (+Ara) and without (-Ara) arabinose induction served as negative controls. Cells were stained with FM-4-64 (red) and Hoechst stain (blue). The micrographs from left to right are FM-4-64, Hoechst, phase contrast and a merged image of the two fluorescent micrographs. Representative images of two independent experiments are shown. Scale bar, 2 µm. (E) Cell length (µm) and (F) Hoechst intensity in cells analyzed in (D). The red line shows the median with interquartile range. The graph shows a combined count from 3 random frames of a representative result, the number of cells (n) per sample is indicated Statistics were performed with data mean ± SEM of three frames. Different letters above the bar indicate statistically different groups of strains ($P < 0.05$) determined by Tukey's HSD test.

Supplementary Table 1. Bacterial strain and plasmids used in this study.

Supplementary Table 2. Primers used in this study.

Figure S1. Genetic loci of *A. tumefaciens* 1D1609 T6SS gene cluster and *vgrG* genetic modules.

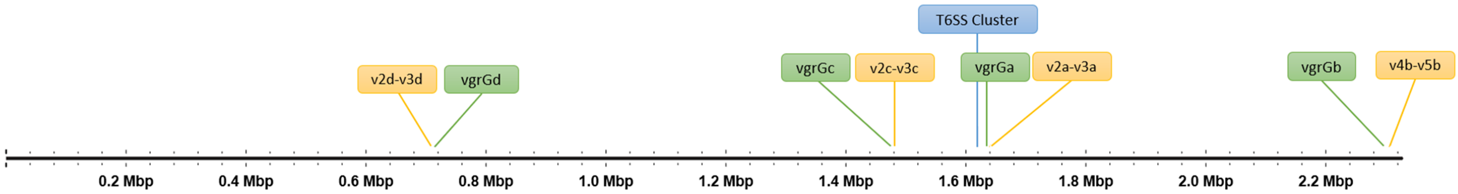

Figure S2. Sequence alignment of V2a and V2c.

```
V2a             MSIPRDNYIGEPQYPGTWTTATPREGLRDIDEARIVSLAPDVCLTPVGSSVVPIPYPVVD
V2c             MSIPRDNYIGEPQYPGTWTTATPREGLRDIDEARIVSLAPDVCLTPVGSSVVPIPYPVVD
                ************************************************************

V2a             FCGHDKNYTPSVRFTGKKAMVMRSFTTHVHGDAPGNRKGVKSGTVESICEPIGHADQVRA
V2c             FCGHDKNYTPSVRFTGKKAMVMRSFTTHVHGDAPGNRKGVKSGTVESICEPIGHADQVRA
                ************************************************************

V2a             EGSHVIRHLDRFYMNSRNTQGEAIFVRSTQTYDPPKDDDPVRGSLRWQDGSDEGRVMSDA
V2c             EGSHVIRHLDRFYMNSRNTQGEAIFVRSTQTYDPPKDDDPVRGSLRWQDGSDEGRVMSDA
                ************************************************************

V2a             SPEPFKEGEKYAYAGSAAAATTGAAQNSAQALRGGMPATRPTVPQTVPRGPGRVITPGPA
V2c             SPEPLIMGAQYAQAQTATIPQSGGV-----ARRGTYGRPTTTVDPRLLKGL-RFWSSARG
                ****:  * :** * :*: . :*..     * **    . .**   : .*  *. :.. .

V2a             RAP---QWYRTPPHRVVSDLA-KVG---------RLARVGIWAR-RLNVIGILSSVLIPD
V2c             AAEIMDMVQQISEDRAVQDSAGQLGLNLSDPTDVLVARAHAWSKYHIDASPFYWD--FPN
                 *       . .  *.*.* * ::*          :**.  *:. .::.  :  .  :*:

V2a             NGANLSDVVPRDDQERRIKEMFDEGVDAGHDSWEMRDWAMEELRRHRQRVREEPESRPQA
V2c             SQ------VGRDAATKAITSM--EAAAPG----TLGE-ALQGDRTQLEKLRTAIKNAVSA
                .       * **   . *..*  *.. .*     : : *::  * : :.:*   :.  .*

V2a             LPQSETVRVSDRDEYRKKCQVDRYGKMKSVCGQYGMQAHHIVPDWTLRYGAREEADKRIP
V2c             ATAAASVRVSG-----QPCAREALG----APGKYRGGAHFAT---ALPLGDNLDSHHTPA
                 . : :****.     : *  :  *    . *:*   **. .   :*  * . :: :  .

V2a             NMPSLNDGMAICVIGNARVEQTEHWGGHLAD------KAIENAGRNSTPPFTAPLHKVVQ
V2c             NSASY----------------LPTWAGPAIQMLPEDHRATLSYGRKVTSPYISTQARLIN
                * .*                    *.*   :      .*  . **: *.*: :.  .:::

V2a             ASAQAMISVRPDCAKEIMAALTTQYAGKNPFQLLRAK--QYPPLPEETISALKSGAVEGA
V2c             SG-------------QFMKAINMDILDIKRIAMLRGDPTRYDAAIAQMMAYAECLKREGI
                :.             ::* *:. :  . : : :**..  .* .   : ::  :.   **

V2a             TRKP
V2c             IK--
                 .
```

Figure S3. Cell length analysis of *E. coli* DH10B cells expressing *v2c* catalytic site variants.

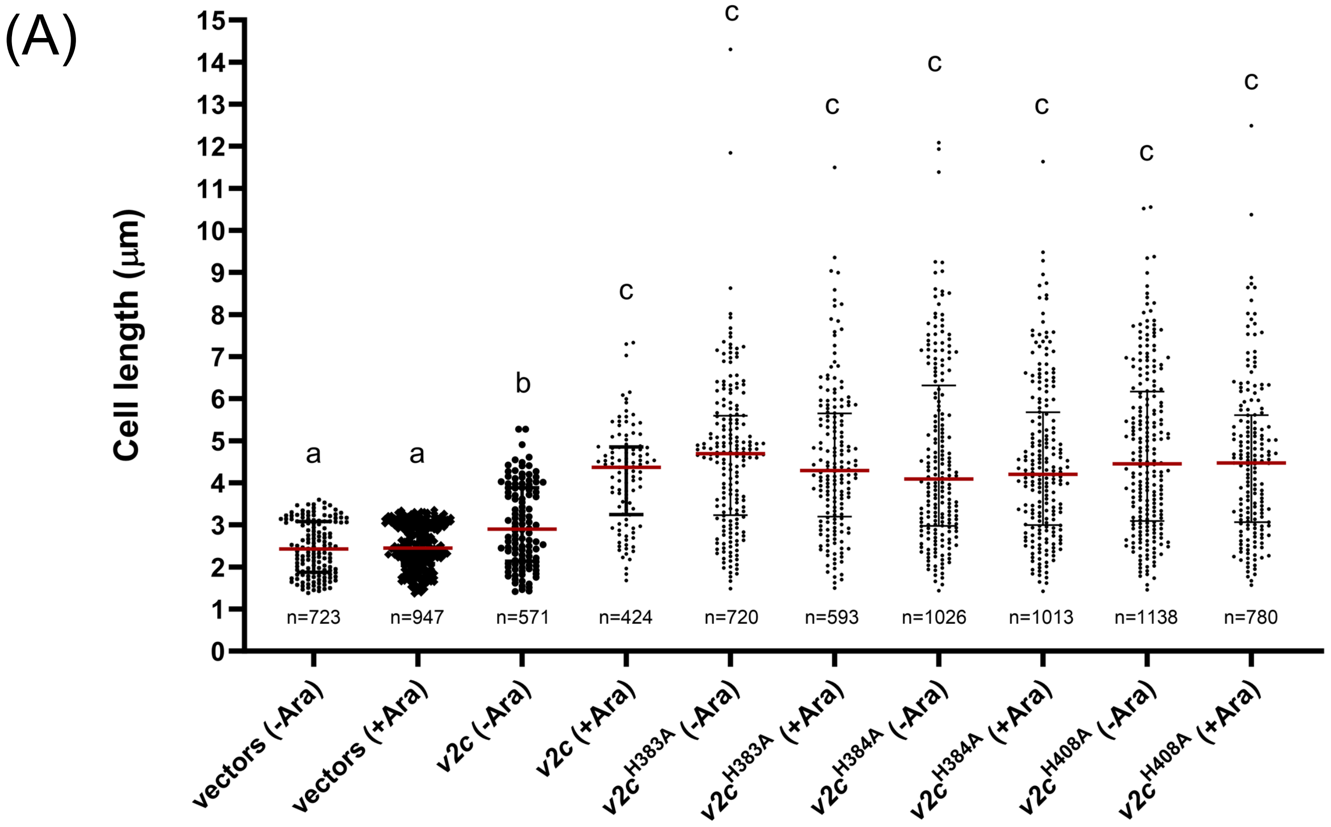

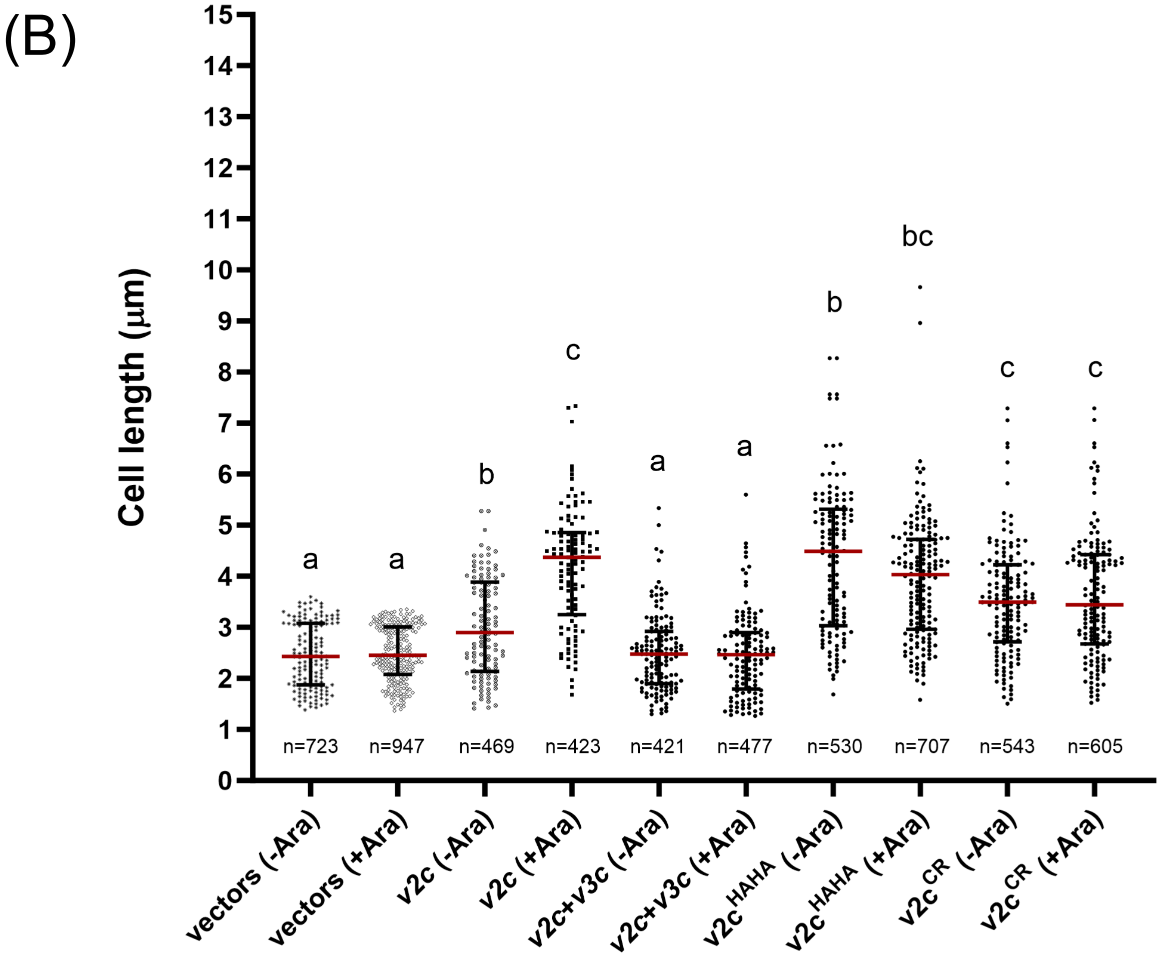

Figure S4. V4b exhibits growth inhibition, which could be neutralized by V5b.

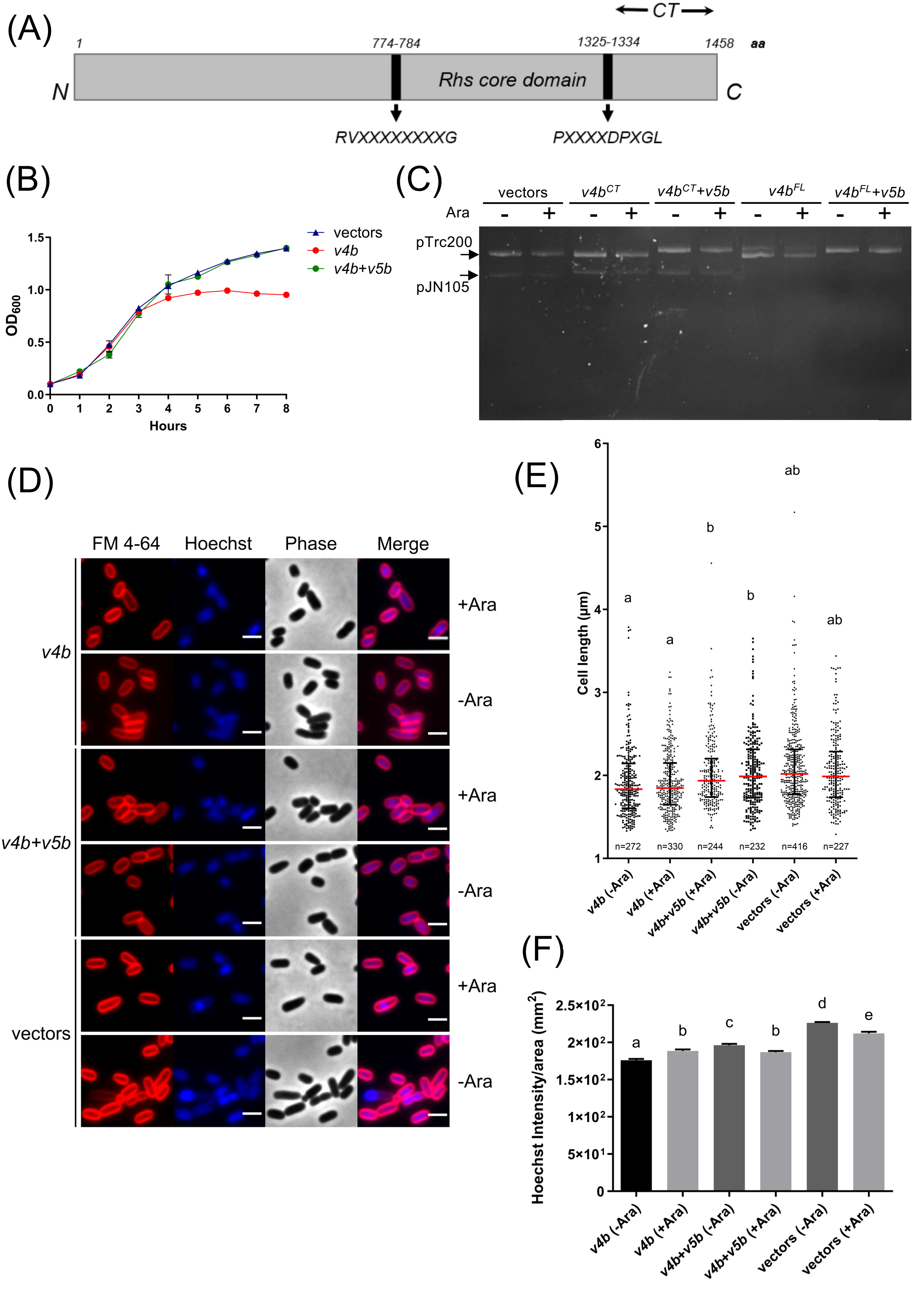

Table S1. Bacterial strains and plasmids

Strain	Relevant characteristics	Reference/Source	EML No.
E. coli			
DH10B	Host of plasmid for cloning	Lab stock	EML455
S17-1	Host for conjugation	Lab stock	EML124
BL21 (DE3)	For protein expression	Lab stock	EML117
***D. dadantii* 3937**			
WT	Wild-type *Dickeya dadantii* 3937	Ching-Hong Yang, University of Wisconsin, Milwaukee	EML4911
Δ*imp*	T6SS inactive strain with deletion of *imp* operon	Ching-Hong Yang, University of Wisconsin, Milwaukee	EML4921
***A. tumefaciens* 1D1609**			
WT	Alfalfa isolate, virulent strain containing pTi1D1609	Clarence Kado, UC Davis	EML304
Δ*4* or Δ*vgrGabcd*	Deletion of *vgrGa*, *vgrGb*, *vgrGc* and *vgrGd*	Santos et al., 2020	EML4746
Δ2*EIbd*	Deletion of *vgrGb*- and *vgrGd*-associated effector-immunity pair	This study	EML5531
Δ*3EIbcd #1*	Deletion of *vgrGb*-, *vgrGc*- and *vgrGd*-associated effector -immunity pair using Δ*2EIbc* as background.	This study	EML5594

Δ*3EIbcd #2*	Deletion of *vgrGb*-, *vgrGc*- and *vgrGd*-associated effector - immunity pair using Δ*2EIcd* as background.	This study	EML5920
Δ3*EIabd*	Deletion of *vgrGa*-, *vgrGb*- and *vgrGd*-associated effector - immunity pair	Santos et al., 2020	EML5515
Δ4*EIabcd*	Deletion of *vgrGa*-, *vgrGb*- and *vgrGd*-associated effector - immunity pair and whole *vgrGc* cluster (Δ*vgrGc*-*v3*)	Santos et al., 2020	EML5540

**Plasmids**			
pJQ200KS	Gm^R, suicide plasmid containing Gm^R and *sacB* gene for selection of double crossover	Quandt and Hynes, 1993	EML121
pRL662	Gm^R, broad host range vector derived from pBBR1MCS-2	Vergunst et al., 2000	EML315
pTrc200	Sp^R, pVS1 origin, *lacIq*, *trc* promoter expression vector	Schmidt-Eisenlohr et al., 1999	EML904
pJN105	Gm^R, arabinose-inducible gene expression vector derived from pBBRMCS-1, *araC*-P_{BAD}	Newman and Fuqua, 1999	EML4220
pET28a	Km^R, N-terminal His-tagged protein expression vector	Novagen	EML2485
pJN-*v2a*	Gm^R, pJN105 expressing *v2a*	Santos et al., 2020	EML5272
pJN-*v4b*^{FL}	Gm^R, pJN105 expressing the full-length *v4b*	This study	EML4758

pJN-*v4b*[CT]	Gm^R, pJN105 expressing the C-terminal toxin domain of *v4b*	This study	EML4756
pJN-*v2c*	Gm^R, pJN105 expressing *v2c*	This study	EML5328
pTrc-*v5b*	Sp^R, pTrc200 expressing the immunity protein V5b	This study	EML4759
pTrc-*v3c*	Sp^R, pTrc200 expressing the immunity protein V3c	This study	EML5337
pJN-*v2a*[H385A]	Gm^R, pJN105 expressing *v2a* with amino acid substitution (H385A)	Santos et al., 2020	EML5298
pJN-*v2c*[H383A]	Gm^R, pJN105 expressing *v2c* with amino acid substitution (H383A)	This study	EML5925
pJN-*v2c*[H384A]	Gm^R, pJN105 expressing *v2c* with amino acid substitution (H384A)	This study	EML5926
pJN-*v2c*[H408A]	Gm^R, pJN105 expressing *v2c* with amino acid substitution (H408A)	This study	EML5927
pJN-*v2c*[HAHA]	Gm^R, pJN105 expressing *v2c* with amino acid substitution (H383AH384A)	This study	EML5922
pJN-*v2c*[CR]	Gm^R, pJN105 expressing *v2c* with deletion of the conserve region of Tox-SSH domain	This study	EML5589
pJQ200sk-*v4v5b*	Gm^R, pJQ200sk derived plasmid to generate Δ3*EIbcd* #2	This study	EML5307
pJQ200sk-*v2v3d*	Gm^R, pJQ200sk derived plasmid to generate Δ2*EIbd*, Δ3*EIbcd* #1 and Δ3*EIabd*	This study	EML5340

pRL662-GFP(S65T)	GFP from pRU1156 cloned into pRL662	Lab stock	EML3376
pET28a-*v2c*	pET28a expressing N-terminal tagged His-V2c	This study	EML6050
pET28a-*v2c*^{HAHA}	pET28a expressing N-terminal tagged His-V2c^{H383AH384A}	This study	EML6051
pET28a-*v2c*^{H408A}	pET28a expressing N-terminal tagged His-V2c^{H408A}	This study	EML 6063
pET28a-*v2c*^{H383A}	pET28a expressing N-terminal tagged His-V2c^{H383A}	This study	EML 6064
pET28a-*v2c*^{H384A}	pET28a expressing N-terminal tagged His-V2c^{H384A}	This study	EML 6065

Table S2. Primers used in this study

Primer Name	Sequences	Purpose
V2c(H383A)	5'-CCGGAATTCAAGGAACAGCTATGAGCATCCCCCGCGAC-3’<br>5'- GACAATTTAGATAGCGCGCATACTCCTGCTAACTCT-3'<br>5'- AGAGTTAGCAGGAGTATGCGCGCTATCTAAATTGTC-3'<br>5'- CGTCTAGATCACTTTATGATTCCTTCGCG-3'	mutagenesis
V2c(H384A)	5'-CCGGAATTCAAGGAACAGCTATGAGCATCCCCCGCGAC-3’<br>5'-GACAATTTAGATAGCCATGCGACTCCTGCTAACTCT-3'<br>5'-AGAGTTAGCAGGAGTCGCATGGCTATCTAAATTGTC-3'<br>5'- CGTCTAGATCACTTTATGATTCCTTCGCG-3'	mutagenesis
V2c(H408A)	5'-CCGGAATTCAAGGAACAGCTATGAGCATCCCCCGCGAC-3’<br>5'- ATGCTTCCAGAAGACGCGCGAGCTACACTCTCATAT -3'<br>5'-TGAGAGTGTAGCTCGCGCGTCTTCTGGAAGCAT-3'<br>5'- CGTCTAGATCACTTTATGATTCCTTCGCG-3'	mutagenesis
V2c(CR)	5'-GTTTTTTTGGGCTAGCGAAGGAACAGCTATGAGCATC-3’<br>5'-CCTTTCTTCCATATGAGAGTGTAGCATTGTCGCCAAGTGGGAGGGCCGTC-3'<br>5'-GACGGCCCTCCCACTTGGCGACAATGCTACACTCTCATATGGAAGAAAGG -3'<br>5'-GCGGTGGCGGCCGCTTCACTTTATGATTCCTTCGC-3'	mutagenesis
V2c(H383A H384A)	5'-CCGGAATTCAAGGAACAGCTATGAGCATCCCCCGCGAC-3’<br>5'-GACAATTTAGATAGCCATGCGACTCCTGCTAACTCT-3'<br>5'-AGAGTTAGCAGGAGTCGCATGGCTATCTAAATTGTC-3'<br>5'- CGTCTAGATCACTTTATGATTCCTTCGCG-3'	mutagenesis
pJQ200KS-*v4bv5b*	5'-TTTCTAGACATCTGTTGTTCGCGGCCGATG-3’<br>5'-GCGGATCCATCACCCGCCATGTGAGGCCT-3'<br>5'-GCGGATCCTTCGCTGCCCTGTGACCGTTCT-3'<br>5'-GCCCCGGGGCAAGATGATTCCGGGAATTGAC-3'	deletion mutant
pJQ200KS-*v2dv3d*	5'-GCTCTAGAGAGCGCCGAAATCCGCTT-3'<br>5'-CGGGATCCGATGCTCATGCTGTCTCCCTCTC-3'<br>5'-CGGGATCCGCCAGAAAGCCATAGATTGTCG-3'<br>5'-TCCCCCCGGGGCATCACCACACTCTCGAGCCT-3'	deletion mutant

V4b	5'-CCGTCTAGAAAGGAACAGCTATGGCGGGTGAT-3'<br>overexpression<br>5'-CGGAGCTCGTTTCGATACTCAGCATCGACCG-3'	
V5b	5'-TATAGGTACCCGAGAATAAGAACCGCGTTC-3'<br>overexpression<br>5'-GCTCTAGAATTGGCGAGGCTACAGCG-3'	
V2c	5'-CCGGAATTCAAGGAACAGCTATGAGCATCCCCCGCGACAATTAT-3'<br>overexpression<br>5'-CGTCTAGATCACTTTATGATTCCTTCGCGTTTAAGGC-3'	
V3c	5'-ATCCGAGCTCGTGACAGACAAGTCGGGAGCG-3'<br>overexpression<br>5'-GCTCTAGACCTAGATCCATCGCTTACTGCCTC-3'	

pET28a-*v2c*	5'-AATCTAGAATGAGCATCCCCCGCGACAA-3'.<br>expression<br>5'-AACTCGAGCTTTATGATTCCTTCGC-3'	Protein
pET28a-*v2c*^{HAHA}	5’-GACAATTTAGATAGCGCGGCGACTCCTGCTAACTCT-3’.<br>expression<br>5'-AGAGTTAGCAGGAGTCGCCGCGCTATCTAAATTGTC-3'	Protein
pET28a-*v2c*^{H383A}	5'-GACAATTTAGATAGCGCGCATACTCCTGCTAACTCT-3'<br>expression<br>5'-AGAGTTAGCAGGAGTATGCGCGCTATCTAAATTGTC-3'	Protein
pET28a-*v2c*^{H384A}	5'-GACAATTTAGATAGCCATGCGACTCCTGCTAACTCT-3'<br>expression<br>5'-AGAGTTAGCAGGAGTCGCATGGCTATCTAAATTGTC-3'	Protein
pET28a-*v2c*^{H408A}	5'-ATGCTTCCAGAAGACGCGCGAGCTACACTCTCATAT-3'<br>expression<br>5'-TGAGAGTGTAGCTCGCGCGTCTTCTGGAAGCAT-3'	Protein

Restriction enzyme sites are underlined. UF (upstream forward), UR (upstream reverse), DF (downstream forward) and DR (downstream reverse).
